# Evidence–practice gaps in chronic kidney disease management among patients taking antidiabetic or antihypertensive medications: a nationwide analysis of Japanese primary care electronic health records

**DOI:** 10.1007/s10157-026-02851-8

**Published:** 2026-04-03

**Authors:** Takahiro Imaizumi, Shinji Asada, Hiroki Ono, Sumire Kanai, Shin-ichi Araki

**Affiliations:** 1 Department of Nephrology, Nagoya University Graduate School of Medicine, Nagoya, Japan; 2 Department of Clinical Research Education, Nagoya University Graduate School of Medicine, Nagoya, Japan; 3 Medical Affairs Department, Kyowa Kirin Co., Ltd., Tokyo, Japan; 4 Department of Nephrology, School of Medicine, Wakayama Medical University, Wakayama, Japan

**Keywords:** Chronic kidney disease, Primary care, Quality improvement, Anemia, Electronic health records

## Abstract

**Background:**

The management of chronic kidney disease (CKD) in primary care remains poorly defined despite primary care physicians managing a high proportion of patients with hypertension and diabetes. This study examined CKD-related laboratory testing and anemia management patterns in Japanese primary care settings.

**Methods:**

Using the Japan Medical Data Survey (JAMDAS) database, we analyzed electronic health records from Japanese primary care clinics. We included patients aged ≥ 18 years who received antihypertensive or antidiabetic medications at least twice in 2024 and visited clinics quarterly, excluding those who received kidney replacement therapy.

**Results:**

Among 859,044 eligible patients (mean age: 70.3 years; men: 48.9%), estimated glomerular filtration rate, dipstick proteinuria, serum potassium, and hemoglobin were measured in 76.2%, 11.2%, 62.9%, and 69.6% of patients, respectively. Proteinuria testing declined with increasing age. Of 604,936 patients who underwent any anemia-related laboratory testing, only 5.1%, 0.4%, and 15.1% had their serum ferritin, transferrin saturation, and serum iron measured, respectively. Among 5219 patients with coded renal anemia, 73.9% received erythropoiesis-stimulating agents (ESAs) or hypoxia-inducible factor prolyl hydroxylase inhibitors (HIF-PHIs). Notably, 58.6% of 553 patients with a renal anemia diagnosis and hemoglobin levels ≥ 13 g/dL were treated with ESAs or HIF-PHIs.

**Conclusion:**

Evidence–practice gaps in CKD management were identified in Japanese primary care settings, including limited proteinuria testing and raising hemoglobin levels above guideline-recommended limits, despite inadequate evaluation of iron parameters. These findings indicate that systematic quality improvement initiatives are needed to improve CKD management in primary care.

**Supplementary Information:**

The online version contains supplementary material available at 10.1007/s10157-026-02851-8.

## Introduction

Although evidence-based guidelines for chronic kidney disease (CKD) management are well established, and effective interventions are available when CKD is appropriately diagnosed, a considerable gap persists between recommended practices and actual clinical implementation [1–4]. Novel pharmacological therapies [5–7] and multidisciplinary care models [3, 4] have effectively slowed CKD progression and prevented cardiovascular events, particularly among individuals with hypertension and diabetes mellitus (DM), which increasingly account for kidney failure with replacement therapy (KFRT) [8]. However, access to these treatments remains limited [9], largely because many individuals with CKD remain undiagnosed [10]. This diagnostic gap represents a major barrier to delivering the proven benefits of contemporary CKD management. Inadequate implementation of diagnostic laboratory testing has been identified as a major contributor to this gap [11].

Given this diagnostic challenge, primary care settings become particularly critical. Since most CKD arises from common lifestyle-related diseases, primary care settings serve as the frontline for early detection, diagnosis, and initiation of kidney-protective therapy [12]. Practice patterns within primary care, therefore, play a crucial role in determining whether patients ultimately receive appropriate and timely CKD care. However, previous large-scale database studies have predominantly relied on hospital-based data [13], providing insufficient characterization of real-world testing behaviors in primary care. The widespread adoption of electronic health record systems in Japanese healthcare offers an unprecedented opportunity to gather real-world evidence on primary care practice patterns [14]. Quantifying actual testing implementation proportions is essential for establishing baseline data for targeted quality improvement initiatives.

To address these implementation gaps, proactive screening by healthcare providers is critical for early CKD detection, timely diagnosis, and greater patient awareness [11, 15]. Even trace proteinuria detected by dipstick testing is strongly associated with increased risk of kidney and cardiovascular events [16–18]. However, clinical recognition of these early findings remains insufficient. A 2024 Japanese database study reported that over 90% of patients with an estimated glomerular filtration rate (eGFR) < 90 mL/min/1.73 m^2^ did not undergo quantitative proteinuria assessment, and approximately 40% underwent no proteinuria testing of any kind [13]. The diagnostic impact was substantial: CKD was diagnosed in 5.9% of patients without proteinuria testing and 43.5% of those receiving quantitative testing, underscoring the importance of implementing systematic testing.

Beyond diagnostic challenges, similar evidence-practice gaps are observed in the management of CKD-related complications. For renal anemia, clinical guidelines strongly recommend iron studies before initiating oral hypoxia-inducible factor prolyl hydroxylase inhibitors (HIF-PHIs), which have become increasingly used in primary care because of their oral administration [19]. However, concerns about thromboembolic risk necessitate appropriate iron parameter monitoring [20]. Evidence–practice gaps like those seen with urinalysis implementation may also apply to iron assessment practices; however, data examining these patterns in primary care settings remain limited [21].

In this study, we aimed to quantify evidence–practice gaps in CKD management within Japanese primary care by evaluating both urinalysis implementation among patients with lifestyle-related diseases and iron assessment practices in renal anemia management, using large-scale primary care EHR data. By quantifying deviations from current guideline recommendations, we sought to clarify evidence–practice gaps in CKD management within Japanese primary care and to inform targeted quality improvement strategies.

## Material and methods

### Study design and data source

Using the Japan Medical Data Survey (JAMDAS) database (M3, Inc., Japan), we conducted a descriptive observational study. Data were extracted on March 7, 2025. The database comprises nationwide electronic health records (EHR) from Japanese general practitioners and primary care clinics [14], including the International Classification of Diseases, tenth revision codes, laboratory test results, medication prescriptions, and clinic locations from participating clinics across Japan. The study protocol was approved by the institutional review board at Kyowa Kirin Co., Ltd. (approval number: MA2024_007_00).

### Study population

From January 1 to December 31 2024, we included patients who met the following criteria: (A) aged ≥ 18 years; (B) received antihypertensive or antidiabetic medications at least twice during the study period; (C) received regular healthcare (at least one clinic visit per quarter); and (D) attended clinics specializing in relevant medical fields, including general internal medicine, metabolic medicine, cardiology, diabetology, nephrology, endocrinology, urology, hemodialysis, and/or dermatourology.

The exclusion criteria included patients receiving kidney replacement therapy (hemodialysis, peritoneal dialysis, or kidney transplantation) and those attending facilities without eGFR testing records throughout the study period. Supplementary Table 1 shows key definitions for inclusion and exclusion: antihypertensive and antidiabetic medications based on ATC codes and diagnosis of KFRT based on reimbursement claim codes.

### Variable definitions

CKD-related laboratory testing included eGFR, dipstick proteinuria, and serum potassium measurements. For patients with multiple laboratory measurements, we used the first available record for each laboratory parameter. eGFR was calculated using the Japanese Society of Nephrology equation when not directly recorded: eGFR (mL/min/1.73m^2^) = 194 × serum creatinine (mg/dL)^−1.094^ × age (years)^−0.287^ × 0.739 (if female). CKD staging followed the kidney disease improving global outcomes guidelines based on eGFR values: G1 (≥ 90), G2 (60–89), G3a (45–59), G3b (30–44), G4 (15–29), and G5 (< 15 mL/min/1.73m^2^).

Anemia medications included iron preparations, ESAs, and HIF-PHIs, identified using ATC codes and reimbursement claim codes (**Supplementary Table 2**). Iron-related laboratory testing included measurements of hemoglobin, ferritin, transferrin saturation (TSAT), serum iron (Fe), and total iron-binding capacity (TIBC). CKD and comorbidity medications included renin–angiotensin system (RAS) inhibitors, sodium–glucose cotransporter-2 (SGLT2) inhibitors, mineralocorticoid receptor antagonists (MRA), and angiotensin receptor–neprilysin inhibitors (ARNI), defined using ATC codes and reimbursement claim codes (Supplementary Table 3).

### Statistical analysis

We performed descriptive analyses, presenting continuous variables as mean (standard deviation) and categorical variables as numbers and percentages. The percentage of patients receiving each laboratory test was calculated as the proportion of patients who had at least one test record during the study period. Anemia treatment patterns, including iron preparations, were analyzed among those who were prescribed any of the following medications: iron preparations, ESAs, or HIF-PHIs. Anemia-related laboratory testing patterns were analyzed among those who were measured any of the following parameters: hemoglobin, ferritin, TSAT, Fe, or TIBC. These two descriptive statistics were summarized according to the CKD stage. Furthermore, treatment patterns with ESAs or HIF-PHIs were analyzed among patients with renal anemia (diagnosis code: 2858001) and those with hemoglobin measurements. To assess the comprehensiveness of renal anemia diagnosis and management, we applied four hierarchical definitions: (1) diagnosis code recorded; (2) diagnosis code with ESA or HIF-PHI treatment; (3) diagnosis code with available hemoglobin measurement; and (4) diagnosis code with both treatment and hemoglobin measurement.

Subgroup analyses were conducted by age group (≤ 44, 45–64, 65–74, 75–84, 85–94, ≥ 95 years), CKD stage, and geographical region (Hokkaido/Tohoku, Kanto, Tokyo, Chubu, Kinki, Chugoku/Shikoku, Kyushu/Okinawa). All analyses were conducted using GoogleSQL, Microsoft Excel 2016, and OriginPro2024 (OriginLab Corp., MA, USA).

## Results

### Study population, demographic data, and practice patterns

**Fig. 1:**
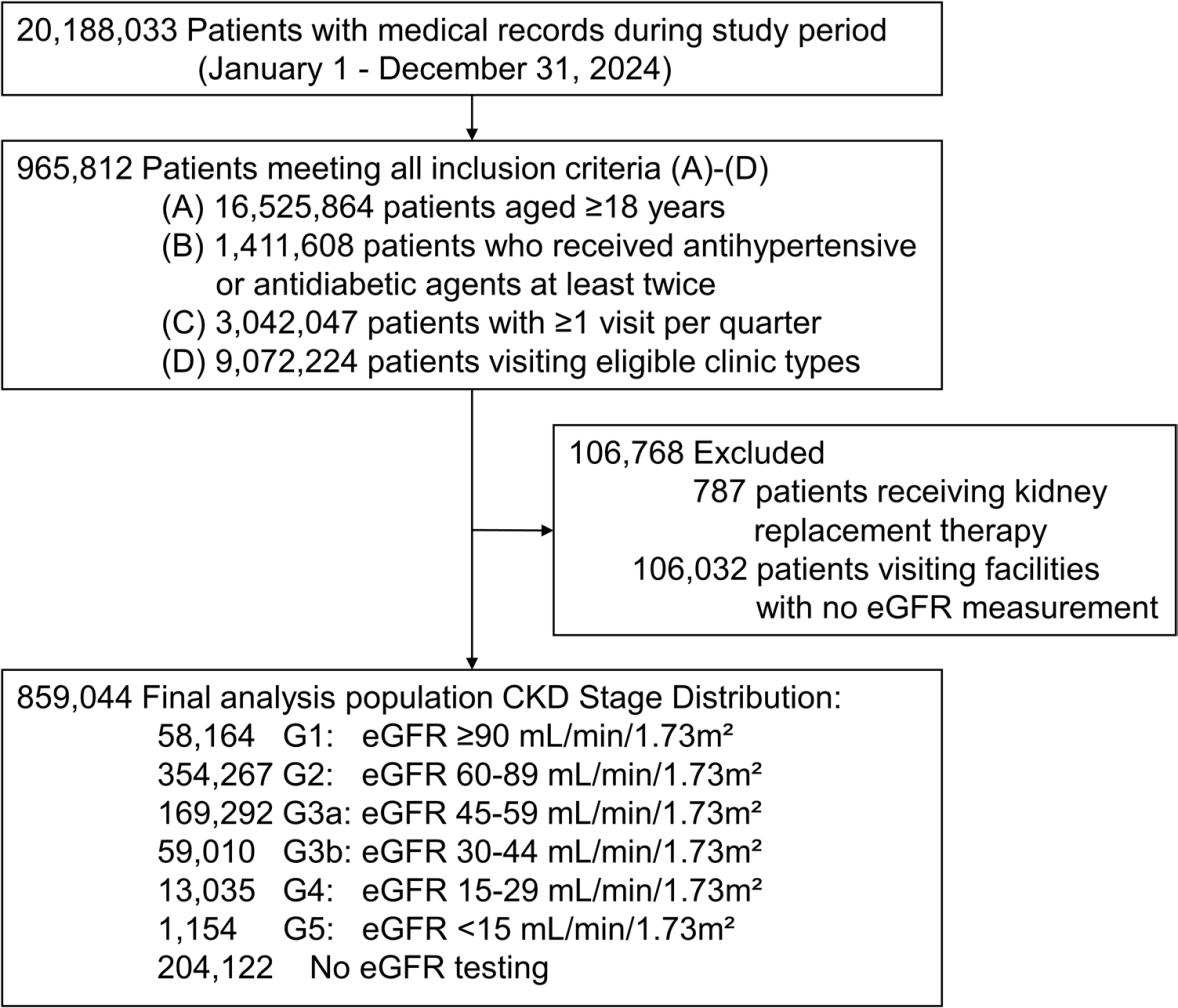
Flow diagram of study population selection

Among 20,188,033 patients in the JAMDAS database in 2024, we identified 859,044 patients who met the inclusion criteria (mean age: 70.3 (13.3) years; men: 419,695 (48.9%); Fig. 1). Common comorbidities included hypertension (754,783, 87.9%), DM (337,902, 39.3%), and dyslipidemia (504,372, 58.7%). Renal anemia was coded in 9230 (1.1%) patients. RAS inhibitors were the most frequently prescribed treatment (484,799, 56.4%), followed by SGLT2 inhibitors (129,851, 15.1%) (Table 1).

**Table 1:** Baseline characteristics and laboratory testing implementation in primary care patients (*n* = 859,044)

	*n* = 859,044	
Sex (male)	419,695	(48.9%)
Age, years	70.3	(13.3)
≤ 44	28,979	(3.4%)
45–64	241,813	(28.1%)
65–74	222,933	(26.0%)
75–84	246,803	(28.7%)
85–94	107,977	(12.6%)
≥ 95	10,539	(1.2%)
*Comorbidities*		
Hypertension	754,783	(87.9%)
Diabetes mellitus	337,902	(39.3%)
Dyslipidemia	504,372	(58.7%)
Renal anemia	9230	(1.1%)
*Proportion receiving laboratory tests*		
eGFR	654,922	(76.2%)
Dipstick proteinuria	96,093	(11.2%)
Serum potassium	540,468	(62.9%)
Hemoglobin	597,819	(69.6%)
Ferritin	30,742	(3.6%)
TSAT	2132	(0.2%)
Fe	91,250	(10.6%)
TIBC	21,626	(2.5%)
*Medication*		
RAS inhibitors	484,799	(56.4%)
SGLT2 inhibitors	129,851	(15.1%)
MRA	48,132	(5.6%)
ARNI	40,182	(4.7%)
Iron preparation	24,068	(2.8%)
ESA	2286	(0.3%)
HIF-PHI	4602	(0.5%)
*Laboratory result categories*		
eGFR, mL/min/1.73 m^2^		
≥ 90	58,164	(6.8%)
60–89	354,267	(41.2%)
45–59	169,292	(19.7%)
30–44	59,010	(6.9%)
15–29	13,035	(1.5%)
< 15	1154	(0.1%)
No result	204,122	(23.8%)
*Dipstick proteinuria*		
−	72,367	(8.4%)
±	11,407	(1.3%)
1 + or above	12,319	(1.4%)
No result	762,951	(88.8%)
*Hemoglobin, g/dL*		
< 10	11,577	(1.3%)
10–12.9	196,698	(22.9%)
≥ 13	389,544	(45.3%)
No result	261,225	(30.4%)

Summarized as mean (standard deviation) for continuous variables and numbers (percentage) for categorical variables

*ARNI* angiotensin receptor–neprilysin inhibitors, *eGFR* estimated glomerular filtration rate, *ESA* erythropoiesis-stimulating agents, *HIF-PHI* hypoxia-inducible factor prolyl hydroxylase inhibitors, *MRA* mineralocorticoid receptor antagonists, *RAS* renin–angiotensin system, *TIBC* total iron-binding capacity, *TSAT* transferrin saturation, *SGLT2* sodium–glucose cotransporter-2

### Proportion receiving CKD-related laboratory tests

Implementation of laboratory testing for the diagnosis and classification of CKD revealed significant disparities. eGFR was measured in 654,922 (76.2%) patients, whereas dipstick proteinuria testing was performed in 96,093 (11.2%) patients. Among 859,044 eligible patients, 58,164 (6.8%), 354,267 (41.2%), 169,292 (19.7%), 59,010 (6.9%), 13,035 (1.5%), and 1,154 (0.1%) had G1, G2, G3a, G3b, G4, and G5 CKD, respectively. Additionally, 72,367 (8.4%), 11,407 (1.3%), and 12,319 (1.4%) had a negative, trace, and positive proteinuria result, respectively (Table 1). The proportion of patients whose CKD stage was determined based on eGFR, and dipstick urinalysis data are shown in Supplementary Table 4. Overall, urinalysis data were not available in 768,350 patients (89.4%).

**Fig. 2:**
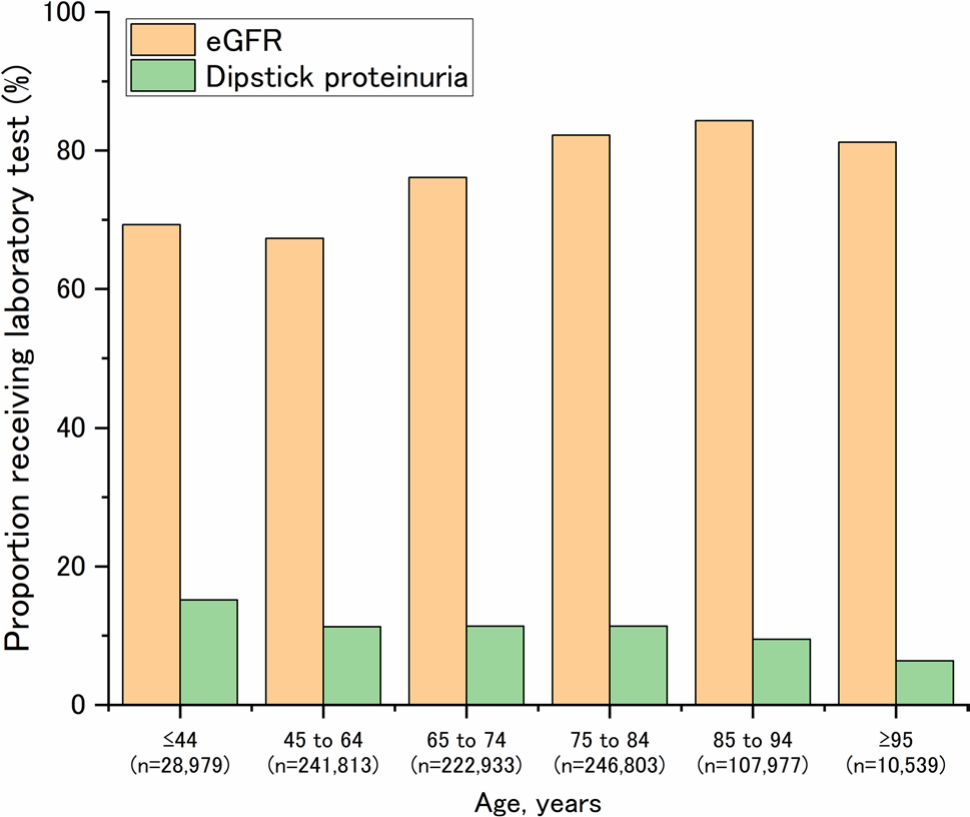
Age-stratified laboratory testing implementation in primary chronic kidney disease management. eGFR levels were more likely to be measured in older adult patients, whereas dipstick urine testing was less frequently performed in this population. eGFR, estimated glomerular filtration rate

Patients receiving proteinuria tests remained consistently low across all stages (13.2%–16.6%) (Table 2). Patients who received dipstick proteinuria tests varied substantially based on age, with the highest and lowest proportions in middle (≤ 44 years: 15.2%) and older age groups (85–94 years: 9.5%; ≥ 95 years: 6.4%), respectively. Conversely, the proportion who measured eGFR increased with age (Fig. 2). Regarding regional practice variations, serum potassium and eGFR measurements were almost consistent across regions, whereas substantial variations in dipstick proteinuria measurements were observed across geographic regions (Supplementary Table 5). The proportion of patients who received dipstick proteinuria tests was the highest in the Chubu region (15.5%), followed by Tokyo (13.5%) and Kanto, excluding Tokyo (13.4%).

**Table 2:** CKD stage-stratified analysis of laboratory testing implementation and treatment patterns

	G1 (*n* = 58,164)	G2 (*n* = 354,267)	G3a (*n* = 169,292)	G3b (*n* = 59,010)	G4 (*n* = 13,035)	G5 (*n* = 1154)	Not available (*n* = 204,122)
Sex (male)	29,685 (51.0%)	174,308 (49.2%)	78,149 (46.2%)	25,678 (43.5%)	5335 (40.9%)	559 (48.4%)	105,981 (51.9%)
Age, years	60.1 (15.3)	68.9 (12.3)	76.1 (10.3)	81 (9.4)	83 (9.8)	79.4 (12.2)	66.8 (13.3)
*Comorbidities*							
Hypertension	43,078 (74.1%)	305,299 (86.2%)	153,522 (90.7%)	54,353 (92.1%)	12,083 (92.7%)	1057 (91.6%)	185,391 (90.8%)
Diabetes mellitus	33,381 (57.4%)	152,556 (43.1%)	67,949 (40.1%)	26,205 (44.4%)	6465 (49.6%)	636 (55.1%)	50,710 (24.8%)
Dyslipidemia	34,435 (59.2%)	221,216 (62.4%)	109,428 (64.6%)	37,785 (64%)	8144 (62.5%)	701 (60.7%)	92,663 (45.4%)
*Laboratory tests*							
Dipstick proteinuria	9629 (16.6%)	48,682 (13.7%)	22,564 (13.3%)	7939 (13.5%)	1725 (13.2%)	155 (13.4%)	5399 (2.6%)
Serum potassium	47,438 (81.6%)	285,723 (80.7%)	139,827 (82.6%)	51,976 (88.1%)	12,091 (92.8%)	1085 (94.0%)	2328 (1.1%)
*Medication*							
RAS inhibitors	26,772 (46.0%)	194,404 (54.9%)	101,501 (60.0%)	36,662 (62.1%)	7686 (59.0%)	604 (52.3%)	117,170 (57.4%)
SGLT2 inhibitors	17,937 (30.8%)	55,694 (15.7%)	23,749 (14.0%)	13,433 (22.8%)	3895 (29.9%)	274 (23.7%)	14,869 (7.3%)
MRA	2087 (3.6%)	15,201 (4.3%)	12,365 (7.3%)	8487 (14.4%)	2736 (21.0%)	164 (14.2%)	7092 (3.5%)
ARNI	1870 (3.2%)	14,044 (4.0%)	9910 (5.9%)	5817 (9.9%)	1918 (14.7%)	149 (12.9%)	6474 (3.2%)

Summarized as mean (standard deviation) for continuous variables and numbers (percentage) categorical variables

*ARNI* angiotensin receptor–neprilysin inhibitors, *MRA* mineralocorticoid receptor antagonists, *RAS* renin–angiotensin system, *SGLT2* sodium–glucose cotransporter-2

Also, serum potassium and hemoglobin, which are important CKD-related laboratory tests, were measured in 540,468 (62.9%) and 597,819 (69.6%) patients, respectively. When stratified by CKD stage, serum potassium and hemoglobin were more likely to be measured in patients with advanced stage CKD.

### Anemia treatment and iron assessment patterns

Among the total population of 859,044, 28,368 (3.3%) patients received any of the following anemia medications: iron preparations, ESAs, or HIF-PHIs. Iron preparations were used most frequently (84.8%), followed by HIF-PHIs (16.2%) and ESAs (8.1%). With advancing CKD stage, the prescription of iron preparations decreased, while that of ESAs or HIF-PHIs increased substantially (Table 3). Notably, a small but non-negligible proportion of patients with eGFR > 60 mL/min/1.73 m^2^ received ESA or HIF-PHI therapy (< 5%), which may represent inappropriate prescribing given their preserved kidney function.

**Table 3:** Anemia treatment and anemia-related parameter monitoring by CKD stage

	Total	G1	G2	G3a	G3b	G4	G5	Not available
Any of the following treatment	*n* = 28,368	*n* = 1862	*n* = 7640	*n* = 6034	*n* = 5688	*n* = 3509	*n* = 543	*n* = 3092
Iron preparation*	24,068 (84.8%)	1850 (99.4%)	7500 (98.2%)	5557 (92.1%)	4285 (75.3%)	1906 (54.3%)	232 (42.7%)	2738 (88.6%)
ESA*	2286 (8.1%)	7 (0.4%)	63 (0.8%)	241 (4.0%)	705 (12.4%)	882 (25.1%)	204 (37.6%)	184 (6.0%)
HIF-PHI*	4602 (16.2%)	14 (0.8%)	161 (2.1%)	548 (9.1%)	1615 (28.4%)	1622 (46.2%)	310 (57.1%)	332 (10.7%)
Any of the following testing	*n* = 604,936	*n* = 53,039	*n* = 321,665	*n* = 153,955	*n* = 54,250	*n* = 12,123	*n* = 1076	*n* = 8828
Hemoglobin**	597,819 (98.8%)	52,551 (99.1%)	318,286 (98.9%)	152,084 (98.8%)	53,386 (98.4%)	11,857 (97.8%)	1043 (96.9%)	8612 (97.6%)
Ferritin**	30,742 (5.1%)	2322 (4.4%)	12,070 (3.8%)	8082 (5.2%)	5195 (9.6%)	2099 (17.3%)	262 (24.3%)	712 (8.1%)
TSAT**	2132 (0.4%)	140 (0.3%)	770 (0.2%)	586 (0.4%)	411 (0.8%)	166 (1.4%)	23 (2.1%)	36 (0.4%)
Fe**	91,250 (15.1%)	7468 (14.1%)	43,021 (13.4%)	24,140 (15.7%)	11,589 (21.4%)	3663 (30.2%)	411 (38.2%)	958 (10.9%)
TIBC**	21,626 (3.6%)	1756 (3.3%)	8774 (2.7%)	5652 (3.7%)	3470 (6.4%)	1369 (11.3%)	174 (16.2%)	431 (4.9%)

*ESA* erythropoiesis-stimulating agent, *HIF-PHI* hypoxia-inducible factor prolyl hydroxylase inhibitor, *TSAT* transferrin saturation, *TIBC* total iron-binding capacity, *Fe* serum iron

*Among patients receiving anemia treatment (Iron preparation, ESA, or HIF-PHI)

**Among patients with anemia-related laboratory tests (Hemoglobin, ferritin, TSAT, Fe, or TIBC)

Hemoglobin was measured in 597,819 (69.6%) patients; however, ferritin, TSAT, Fe, and TIBC were measured only in 30,742 (3.6%), 2,132 (0.2%), 91,250 (10.6%), and 21,626 (2.5%) patients, respectively (Table 1). Among the 604,936 patients who underwent anemia-related testing, the proportions were still low: ferritin in 5.1%, TSAT in 0.4%, and serum iron in 15.1% of patients. These proportions increased as CKD stage advanced; however, ferritin and TSAT were measured in 24.3% and 2.1% of patients with stage 5 CKD (Table 3).

**Fig. 3:**
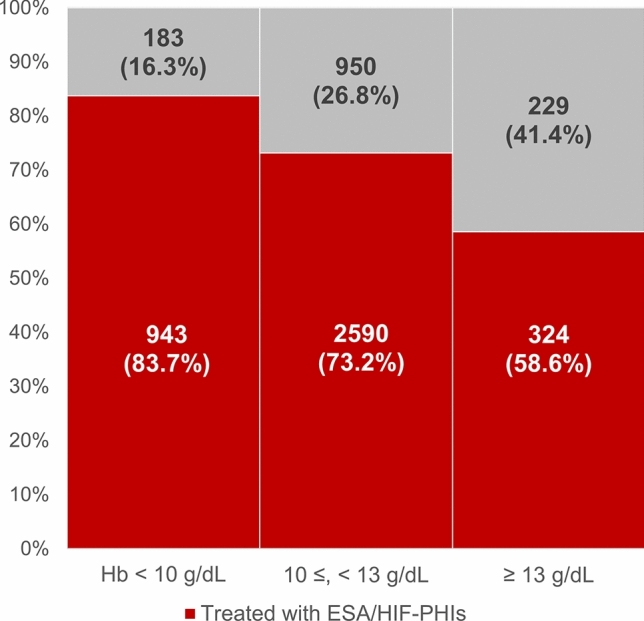
Treatment implementation by hemoglobin levels among patients with coded renal anemia and their hemoglobin values available. Among 5219 patients with diagnostic code for renal anemia and hemoglobin measurements, proportions treated with ESA/HIF-PHIs were 83.7% (< 10 g/dL), 73.2% (10–12.9 g/dL), and 58.6% (≥ 13 g/dL). *Hb* hemoglobin, *ESA* erythropoiesis-stimulating agent, *HIF-PHI* hypoxia-inducible factor prolyl hydroxylase inhibitor

Among patients with a recorded renal anemia diagnosis code (definition 1; n = 9,230), 6,589 (71.4%) had received ESA or HIF-PHI treatment (definition 2), 5,219 (56.5%) had an available hemoglobin measurement (definition 3), and 3,857 (41.8%) fulfilled the most comprehensive definition including both treatment and hemoglobin measurement (definition 4) (Supplementary Table 6). Of these 9,230 patients, 836 (9.1%) lacked eGFR documentation, and among them, only 58 (7.0%) had hemoglobin measurements available. Among 5219 patients with renal anemia diagnosis codes and hemoglobin measurements, 3,857 (73.9%) were given ESAs or HIF-PHIs. Even patients with adequate hemoglobin levels were likely to be prescribed these medications. Among patients with hemoglobin levels ≥ 13 g/dL, 324 (58.6%) received ESAs or HIF-PHIs (Fig. 3). Conversely, among patients with hemoglobin levels < 10 g/dL, 183 (16.3%) were not treated with ESAs or HIF-PHIs. When stratified by CKD stage, patients with more advanced CKD stage were more likely to be prescribed ESA or HIF-PHI, even with hemoglobin levels ≥ 13 g/dL (Supplementary Table 7).

## Discussion

This study is the first comprehensive investigation of CKD management practices in Japanese primary care settings via analyzing a large-scale electronic health record database, particularly focusing on renal anemia management, including evaluation of iron parameters. Our findings reveal substantial evidence–practice gaps in 2024 that have key implications for patient care quality and clinical outcomes. Three major evidence–practice gaps emerged from our analysis. First, 11.2% of patients received dipstick proteinuria testing, whereas 76.2% of patients on antihypertensive or antidiabetic medications underwent annual eGFR measurements, representing a critical diagnostic opportunity gap. Second, despite the fact that iron parameter assessments are essential in renal anemia management, ferritin and TSAT measurements were only tested in 5.1% and 0.4% of patients undergoing anemia-related testing, respectively. Third, among patients with coded renal anemia, 58.6% of those with hemoglobin ≥ 13 g/dL received ESA/HIF-PHI treatment. Although our cross-sectional study design does not allow the evaluation of temporal treatment patterns, this finding suggests a critical evidence–practice gap given that the Japanese Society of Nephrology guidelines recommend treatment discontinuation when hemoglobin levels exceed 13 g/dL [1].

The low proportion of urinalysis has been consistently recognized as an issue in Japanese primary care settings. Analysis using Japan’s National Database of Health Insurance Claims and Specific Health Checkups showed that although the proportion of patients with DM who underwent annual HbA1c or glycoalbumin testing was markedly high (96.7%), dipstick urinalysis implementation was 67.3% in facilities with < 200 beds, and quantitative proteinuria testing was performed in 19.4% of patients [22]. Although quantitative proteinuria testing is preferable to avoid missing a substantial proportion of CKD cases [23], dipstick testing has demonstrated favorable cost-effectiveness [24, 25] and represents a practical first step that should be actively implemented. Several factors may contribute to the underutilization of urinalysis. First, despite being less invasive than blood sampling, the observed age-related decline in tested proportions suggests potential barriers related to urine collection, fall risks during sample collection, and insufficient staffing to address these concerns for older adult patients. Second, the low reimbursement for urinalysis provides limited financial incentives for healthcare providers to implement this testing. Given that urinalysis, including qualitative measurements, is cost-effective [26], financial incentives may boost urine testing. Third, awareness of the clinical importance of urinalysis may be insufficient among healthcare providers. Educational initiatives targeting general practitioners are a critical priority for quality improvement. The observed regional variations in dipstick proteinuria testing (ranging from 6.4–15.5%) further highlight the need for standardized quality improvement initiatives and underscore the importance of nationwide educational interventions to ensure equitable access to appropriate CKD screening.

We also identified substantial challenges in renal anemia management among patients with advanced CKD. Although the advent of oral HIF-PHI formulations has facilitated prescriptions in primary care settings compared to conventional ESA agents, evaluating iron parameters has remained inadequate. In our study, TSAT measurement was performed in 0.4% of patients, and ferritin measurement in 5.1% of patients, revealing severe neglect of iron kinetics evaluations. Of particular concern is that HIF-PHI agents carry thrombosis risks, necessitating appropriate iron parameter monitoring [27–30]; however, basic evaluations were not performed in primary care settings. Most importantly, among patients with coded renal anemia, 58.6% of those with hemoglobin ≥ 13 g/dL continued to receive ESA/HIF-PHI treatment. This represents a clear deviation from the Japanese Society of Nephrology guidelines, which recommend treatment discontinuation or dose reduction when hemoglobin levels exceed 13 g/dL, suggesting a potential increased thrombotic risk from overtreatment [1]. Additionally, our findings revealed that a small proportion of patients with preserved kidney function (eGFR > 60 mL/min/1.73 m^2^) received HIF-PHI therapy, which represents another form of potentially inappropriate prescribing. Current guidelines recommend HIF-PHI initiation primarily in patients with advanced CKD stages, and their use in patients with mild or no kidney dysfunction lacks evidence-based justification and may expose patients to unnecessary risks.

Despite the important findings identified in this study, concerns regarding the comprehensiveness of clinical assessment warrant acknowledgment. Approximately 9% of patients with a recorded diagnosis of renal anemia lacked eGFR documentation, and hemoglobin measurements were available in only 7% of this subgroup. Nevertheless, the frequency of ESA and HIF-PHI use in this group was comparable to that observed in patients with G3a CKD, suggesting that these patients carried a clinically meaningful disease burden despite the absence of adequate kidney function assessment. This may be a manifestation of care fragmentation in CKD management within primary care settings. Whether this reflects continuation of specialist-initiated treatment without subsequent reassessment in primary care warrants further investigation. These findings also highlight the importance of integrating laboratory data across care settings. In Japan, test results obtained at specialist institutions may not be accessible to primary care physicians, potentially contributing to the gaps identified in this study. The ongoing development of a nationwide health information infrastructure in Japan, aimed at enabling seamless sharing of clinical data including laboratory results across institutions [31], holds promise for reducing such fragmentation and supporting more comprehensive CKD monitoring in primary care.

The following limitations of our study require acknowledgment. First, our study population was restricted to facilities using a single EHR system, which may limit generalizability to all Japanese primary care settings (approximately 5% of primary care practices across Japan). Additionally, patients with severe CKD who were likely referred to specialists were excluded, potentially limiting the comprehensiveness of our findings. Second, the cross-sectional design precludes the establishment of temporal relationships and causal inference, limiting our ability to distinguish between treatment continuation versus initiation decisions. Furthermore, the simultaneous occurrence of elevated hemoglobin levels and ESA/HIF-PHI prescriptions observed in this study does not necessarily indicate the continuation of inappropriate treatment. Rather, it may reflect the temporal lag between receiving test results and making clinical decisions. Future studies involving longitudinal follow-up are necessary to resolve this issue. Third, misclassification bias may have occurred because laboratory testing was performed at external facilities, potentially underestimating the actual proportions of testing implementation. Misclassification bias may also have occurred because we defined renal anemia as having a diagnosis code of 2,858,001. Fourth, as a descriptive study, we did not perform multivariable analysis to adjust for potential confounding factors, such as patient demographics, comorbidity burden, and healthcare accessibility variables, which could influence testing and treatment patterns. Fifth, our study focused specifically on CKD patients receiving antihypertensive or antidiabetic medications, which represents the predominant CKD population in primary care settings but systematically excludes patients with primary glomerulonephritis, autoimmune kidney disease, or early-stage CKD without these comorbidities. This selection strategy prioritizes the most common CKD etiologies in primary care but limits generalizability to the broader CKD population. Sixth, urine dipstick testing results without formal test orders may not be captured as structured data, potentially underestimating actual testing frequency. Finally, our findings reflect the Japanese healthcare system and reimbursement structure, limiting generalizability to other healthcare settings.

This study identified critical evidence–practice gaps in CKD management within Japanese primary care. The current patterns of care, characterized by limited urinalysis implementation (11.2%), insufficient evaluation of iron parameters, and treatment continuation above guideline-recommended hemoglobin targets, suggest opportunities for improving optimal patient care delivery. Future prospective studies should investigate the associations between these practice patterns and clinical outcomes, including thromboembolic events, CKD progression, and cardiovascular complications. Additionally, developing and evaluating comprehensive quality improvement strategies, including educational interventions for primary care physicians, clinical decision support systems, and healthcare reimbursement policy considerations, may improve CKD management in Japan.

## Supplementary Information

Below is the link to the electronic supplementary material.

Supplementary file1 (DOCX 54 KB)
