# Supplementary material for: Evidence–practice gaps in chronic kidney disease management among patients taking antidiabetic or antihypertensive medications: a nationwide analysis of Japanese primary care electronic health records
Source: Clin Exp Nephrol. 2026 Apr 3;30(6):856–65. doi: 10.1007/s10157-026-02851-8 (PMC13242465; doi:10.1007/s10157-026-02851-8)
Supplement: Supplementary file 1 — Supplementary file1 (DOCX 54 KB) [file 10157_2026_2851_MOESM1_ESM.docx]

**Evidence-Practice Gaps in Chronic Kidney Disease Management** **among Patients Taking Antidiabetic or Antihypertensive Medications: A Nationwide Analysis of Japanese Primary Care Electronic Health Records**

**Authors:**

Takahiro Imaizumi*, Shinji Asada, Hiroki Ono, Sumire Kanai, Shin-ichi Araki

***Corresponding author:** Takahiro Imaizumi

[imaizumi.takahiro.r7@f.mail.nagoya-u.ac.jp](mailto:imaizumi.takahiro.r7@f.mail.nagoya-u.ac.jp)

**Supplementary Material**

[**Supplementary Table 1.** Definition of medication and kidney failure with replacement therapy 2](#_Toc222318641)

[**Supplementary Table 2**. Anemia treatment medications: iron preparations, ESAs, and HIF-PHIs 3](#_Toc222318642)

[**Supplementary Table 3**. Definition of CKD-related treatment based on ATC or reimbursement claim codes 4](#_Toc222318643)

[**Supplementary Table 4.** The proportion of patients whose CKD stage was determined based on eGFR and dipstick urinalysis data 6](#_Toc222318644)

[**Supplementary Table 5.** Regional variations in laboratory testing implementation across Japan 7](#_Toc222318645)

[**Supplementary Table 6.** Clinical characteristics of patients with and without eGFR measurements 8](#_Toc222318646)

[**Supplementary Table 7.** CKD stage-stratified ESA/HIF-PHI treatment by hemoglobin levels 9](#_Toc222318647)

**Supplementary Table 1.** Definition of medication and kidney failure with replacement therapy

| Type of agents | Classification | Definition (ATC code, reimburse claim code,  or ICD-10 code) |
| --- | --- | --- |
| Antihypertensive agents | Diuretics, MRA | C03 (ATC code) |
|  | β blockers (including αβ blockers) | C07 (ATC code) |
|  | Calcium channel blockers | C08 (ATC code) |
|  | RAS inhibitors (ACE inhibitors, ARB), Direct renin inhibitors, ARNI | C09 (ATC code) |
|  | Others (α blockers, central sympatholytic agents, etc) | C02 (ATC code) |
| Antidiabetic agents | Insulin | A10A (ATC code) |
|  | Biguanides | A10BA (ATC code) |
|  | Sulfonylureas | A10BB (ATC code) |
|  | Combination drugs | A10BD (ATC code) |
|  | α-Glucosidase inhibitors | A10BF (ATC code) |
|  | Thiazolidinediones | A10BG (ATC code) |
|  | DPP-4 inhibitors | A10BH (ATC code) |
|  | GLP-1 receptor agonists | A10BJ (ATC code) |
|  | SGLT2 inhibitors | A10BK( ATC code) |
|  | Others (glinides, imeglimin, tirzepatide, etc.) | A10BX (ATC code) |
| Diagnosis of kidney failure with replacement therapy | Hemodialysis, peritoneal dialysis, kidney transplantation | 150196310, 150338610, 190167970, 113002510, 114009310, 114009410, 114009510, 140057810, 140057910, 140058010, 140059310, 140059410, 140059510, 140058110, 140058210, 140058310, 140058410, 140058510, 140058610, 140036710, 140051010, 140051110, 140052810 (reimburse claim code) |
|  | Post-kidney transplantation status | Z94.0, T86.1 (ICD-10 code) |

Antihypertensive and antidiabetic medications were identified using ATC classification codes. Kidney failure with replacement therapy was defined using Japanese reimbursement claim codes and ICD-10 diagnostic codes for post-transplantation status.

ATC, anatomical therapeutic chemical; ICD-10, international classification of diseases, tenth revision; MRA, mineralocorticoid receptor antagonists; RAS, renin-angiotensin system; ACE, angiotensin-converting enzyme; ARB, angiotensin receptor blockers; ARNI, angiotensin receptor-neprilysin inhibitors; DPP-4, Dipeptidyl peptidase-4; GLP-1, Glucagon-like peptide-1; SGLT2, Sodium-glucose cotransporter-2

**Supplementary Table 2**. Anemia treatment medications: iron preparations, ESAs, and HIF-PHIs

| **Type of agents** | **Classification** | | **Definition (ATC code or reimbursement claim code)** |
| --- | --- | --- | --- |
| Iron preparations | Intravenous iron preparations | Saccharated ferric oxide | 620005208 |
|  |  | Ferric carboxymaltose | 622676601 |
|  |  | Ferric derisomaltose | 622926501, 622926601 |
|  | Oral iron preparations | Dried ferrous sulfate | B03AA07 |
|  |  | Sodium ferrous citrate | B03AA12 |
|  |  | Ferrous fumarate | B03AA02 |
|  |  | Soluble ferric pyrophosphate | 620005924 |
|  | Iron-containing phosphate binders | Ferric citrate hydrate | V03AE08 |
| ESA | Epoetin | | B03XA01 |
|  | Darbepoetin alfa | | B03XA02 |
|  | Epoetin beta pegol | | B03XA03 |
| HIF-PHI | Roxadustat | | B03XA05 |
|  | Daprodustat | | B03XA07 |
|  | Vadadustat | | B03XA08 |
|  | Enarodustat | | 622829501, 622829601 |
|  | Molidustat sodium | | 622854901, 622855001, 622855101, 622855301 |

Medications were identified using ATC codes when available, with Japanese-specific reimbursement claim codes used for agents not yet assigned international ATC classifications. Iron-containing phosphate binders were included as they serve dual purposes for phosphate control and iron supplementation in CKD patients.

ATC, anatomical therapeutic chemical; ESA, erythropoiesis-stimulating agents; HIF-PHI, hypoxia-inducible factor prolyl hydroxylase inhibitors

**Supplementary Table 3**. Definition of CKD-related treatment based on ATC or reimbursement claim codes

| Type of agents | Classification | | Definition (ATC code or reimbursement claim code) |
| --- | --- | --- | --- |
| RAS inhibitors | ACE inhibitors | Captopril | C09AA01 |
|  |  | Enalapril maleate | C09AA02 |
|  |  | Lisinopril hydrate | C09AA03 |
|  |  | Perindopril erbumine | C09AA04 |
|  |  | Benazepril hydrochloride | C09AA07 |
|  |  | Trandolapril | C09AA10 |
|  |  | Delapril hydrochloride | C09AA12 |
|  |  | Temocapril hydrochloride | C09AA14 |
|  |  | Imidapril hydrochloride | C09AA16 |
|  |  | Alacepril | 610422142, 610433069, 612140479, 612140480, 620281201, 620281304, 620281601, 620281801, 620282504, 620282601, 621245901, 621987002, 621992502, 621992602, 622064602, 622096302, 622309900, 622721800, 622721900, 622722000 |
|  | ARB | Losartan potassium | C09CA01 |
|  |  | Valsartan | C09CA03 |
|  |  | Irbesartan | C09CA04 |
|  |  | Candesartan cilexetil | C09CA06 |
|  |  | Telmisartan | C09CA07 |
|  |  | Olmesartan medoxomil | C09CA08 |
|  |  | Azilsartan | C09CA09 |
|  |  | Fixed-dose combinations | C09DA01, C09DA03, C09DA04, C09DA06, C09DA07, C09DB01, C09DB04, C09DB05, C09DB07, C09DX08, 621974501, 621974601,622343001, 622844001, 622844101, 622844901, 622845001, 622850701, 622850801, 622852101, 622852201, 622857601, 622857701, 622858801, 622858901, 622859001, 622859101, 622865601, 622865701, 622866201, 622866301, 622867801, 622867901, 622880301, 622880401 |
| SGLT2 inhibitors | Dapagliflozin propylene glycol hydrate | | A10BK01 |
|  | Empagliflozin | | A10BK03, A10BD19 |
|  | Canagliflozin hydrate | | A10BK02, 622573601 |
|  | Ipragliflozin L-proline | | A10BK05, 622625702 |
|  | Luseogliflozin hydrate | | A10BK07 |
|  | Tofogliflozin hydrate | | 622336801, 622340101 |
| MRA | Spironolactone | | C03DA01 |
|  | Potassium canrenoate | | C03DA02 |
|  | Eplerenone | | C03DA04 |
|  | Esaxerenone | | 622670101, 622670201, 622670301, 622910101, 622910201, 622910301 |
|  | Finerenone | | C03DA05 |
| ARNI | Sacubitril/valsartan sodium hydrate | | C09DX04 |

Fixed-dose combination products containing RAS inhibitors were classified under ARB combinations. Japanese reimbursement claim codes were used for medications not assigned international ATC codes.

ATC, anatomical therapeutic chemical; RAS, renin-angiotensin system; ACE, angiotensin-converting enzyme; ARB, angiotensin receptor blockers; SGLT2, Sodium-glucose cotransporter-2; MRA, mineralocorticoid receptor antagonists; ARNI, angiotensin receptor-neprilysin inhibitors

**Supplementary Table 4.** The proportion of patients whose CKD stage was determined based on eGFR and dipstick urinalysis data

|  | **Total** | **G stage** | | | | | | |
| --- | --- | --- | --- | --- | --- | --- | --- | --- |
| **Urinalysis** |  | **G1** | **G2** | **G3a** | **G3b** | **G4** | **G5** | **Not available** |
| **Total** | 859,044 | 58,164 | 354,267 | 169,292 | 59,010 | 13,035 | 1,154 | 204,122 |
| **-** | 68,360  (8.0%) | 7,392  (12.7%) | 38,914  (11%) | 16,604  (9.8%) | 4,776  (8.1%) | 659  (5.1%) | 15  (1.3%) | - |
| **±** | 10,693  (1.2%) | 1,257  (2.2%) | 5,366  (1.5%) | 2,760  (1.6%) | 1,069  (1.8%) | 229  (1.8%) | 12  (1.0%) | - |
| **≥1+** | 11,641  (1.4%) | 980  (1.7%) | 4,402  (1.2%) | 3,200  (1.9%) | 2,094  (3.5%) | 837  (6.4%) | 128  (11.1%) | - |
| **Not available** | 768,350  (89.4%) | 48,535  (83.4%) | 305,585  (86.3%) | 146,728  (86.7%) | 51,071  (86.5%) | 11,310  (86.8%) | 999  (86.6%) | 204,122 |
| **Male** | 419,695 | 29,685 | 174,308 | 78,149 | 25,678 | 5,335 | 559 | 105,981 |
| **-** | 32,766  (7.8%) | 3,804  (12.8%) | 19,117  (11%) | 7,622  (9.8%) | 2,020  (7.9%) | 199  (3.7%) | 4  (0.7%) | - |
| **±** | 5,941  (1.4%) | 712  (2.4%) | 3,113  (1.8%) | 1,495  (1.9%) | 523  (2%) | 94  (1.8%) | 4  (0.7%) | - |
| **≥1+** | 7,088  (1.7%) | 596  (2%) | 2,712  (1.6%) | 1,968  (2.5%) | 1,237  (4.8%) | 500  (9.4%) | 75 (13.4%) | - |
| **Not available** | 373,900  (89.1%) | 24,573  (82.8%) | 149,366  (85.7%) | 67,064  (85.8%) | 21,898  (85.3%) | 4,542  (85.1%) | 476 (85.2%) | 105,981 |
| **Female** | 439,349 | 28,479 | 179,959 | 91,143 | 33,332 | 7,700 | 595 | 98,141 |
| **-** | 35,594  (8.1%) | 3,588  (12.6%) | 19,797  (11%) | 8,982  (9.9%) | 2,756  (8.3%) | 460  (6%) | 11  (1.8%) | - |
| **±** | 4,752  (1.1%) | 545  (1.9%) | 2,253  (1.3%) | 1,265  (1.4%) | 546  (1.6%) | 135  (1.8%) | 8  (1.3%) | - |
| **≥1+** | 4,553  (1%) | 384  (1.3%) | 1,690  (0.9%) | 1,232  (1.4%) | 857  (2.6%) | 337  (4.4%) | 53  (8.9%) | - |
| **Not available** | 394,450  (89.8%) | 23,962  (84.1%) | 156,219  (86.8%) | 79,664  (87.4%) | 29,173  (87.5%) | 6,768  (87.9%) | 523 (87.9%) | 98,141 |

**Supplementary Table 5.** Regional variations in laboratory testing implementation across Japan

|  | Hokkaido/Tohoku (40,259) | Kanto (186,641) | Tokyo (104,434) | Chubu (190,608) | Kinki (145,588) | Chugoku/Shikoku (65,838) | Kyusyu/ Okinawa (125,676) |
| --- | --- | --- | --- | --- | --- | --- | --- |
| Sex (male) | 18342 (45.6%) | 92383 (49.5%) | 55051 (52.7%) | 93522 (49.1%) | 69264 (47.6%) | 31344 (47.6%) | 59789 (47.6%) |
| Age, years | 70.9 (13.0) | 69.7 (13.3) | 68 (14.2) | 70.6 (13.2) | 71.1 (13.1) | 72.3 (12.6) | 70.6 (13.1) |
| **Comorbidities** |  |  |  |  |  |  |  |
| Hypertension | 36158 (89.8%) | 163354 (87.5%) | 90904 (87%) | 167338 (87.8%) | 127214 (87.4%) | 58503 (88.9%) | 111312 (88.6%) |
| Diabetes mellitus | 15391 (38.2%) | 72076 (38.6%) | 42734 (40.9%) | 76780 (40.3%) | 55822 (38.3%) | 26985 (41%) | 48114 (38.3%) |
| Dyslipidemia | 24449 (60.7%) | 106188 (56.9%) | 63108 (60.4%) | 114367 (60%) | 84390 (58%) | 39754 (60.4%) | 72116 (57.4%) |
| **Proportion receiving laboratory test** | | | | | | | |
| eGFR | 29118 (72.3%) | 141610 (75.9%) | 75968 (72.7%) | 148995 (78.2%) | 113177 (77.7%) | 50019 (76%) | 96035 (76.4%) |
| Dipstick proteinuria | 3714 (9.2%) | 25045 (13.4%) | 14093 (13.5%) | 29501 (15.5%) | 10346 (7.1%) | 4177 (6.3%) | 9217 (7.3%) |
| Serum potassium | 25967 (64.5%) | 110405 (59.2%) | 50962 (48.8%) | 124293 (65.2%) | 98647 (67.8%) | 43352 (65.8%) | 86842 (69.1%) |

Regional classifications follow standard Japanese geographical divisions: Hokkaido/Tohoku (northern regions), Kanto (eastern regions excluding Tokyo metropolitan area), Tokyo (Tokyo metropolitan area), Chubu (central regions), Kinki (Kansai/western regions), Chugoku/Shikoku (western and southern island regions), and Kyushu/Okinawa (southern regions). Data presented as numbers (percentage) for categorical variables and mean (standard deviation) for continuous variables unless otherwise specified.

eGFR, estimated glomerular filtration rate

**Supplementary Table 6.** Clinical characteristics of patients with and without eGFR measurements

|  | **Total** | **G stage** | | | | | | |
| --- | --- | --- | --- | --- | --- | --- | --- | --- |
|  |  | **G1** | **G2** | **G3a** | **G3b** | **G4** | **G5** | **Not available** |
| **Total** | 859044 | 58164 | 354267 | 169292 | 59010 | 13035 | 1154 | 204122 |
| Definition of renal anemia |  |  |  |  |  |  |  |  |
| 1) Diagnosis code recorded | 9230 | 34 | 354 | 1217 | 3191 | 3039 | 559 | 836 |
| 2) Code + treatment | 6589 (71) | 19  (56) | 207 (58) | 765 (63) | 2229 (70) | 2393 (79) | 479 (86) | 497  (59) |
| 3) Code + Hb measured | 5219 (57) | 28  (82) | 230 (65) | 742 (61) | 2013 (63) | 1815 (60) | 333 (60) | 58  (7) |
| 4) Code + treatment + Hb measured | 3857 (42) | 18  (53) | 137 (39) | 471 (39) | 1433 (45) | 1464 (48) | 286 (51) | 48  (6) |

Percentages for definitions 2–4 are calculated among patients with a recorded diagnosis of renal anemia (definition 1, n=9,230). ‘Code’ is defined as having renal anemia code (2858001). ‘Treatment’ is defined as having received ESA or HIF-PHI therapy. eGFR, estimated glomerular filtration rate; Hb, hemoglobin.

**Supplementary Table 7.** CKD stage-stratified ESA/HIF-PHI treatment by hemoglobin levels

|  | Coded renal anemia | Numbers receiving ESA or HIF-PHI in patients with coded renal anemia | | | | | |
| --- | --- | --- | --- | --- | --- | --- | --- |
|  |  | Hb <10 g/dL  (n=1,126) | | Hb 10–12.9 g/dL (n=3,540) | | Hb ≥13 g/dL  (n=553) | |
|  | (n=5219) | N | Treated | N | Treated | N | Treated |
| G1 | 28 | 5 | 5 (100%) | 17 | 11 (64.7%) | 6 | 2 (33.3%) |
| G2 | 230 | 44 | 29 (65.9%) | 145 | 91 (62.8%) | 41 | 17 (41.5%) |
| G3a | 742 | 122 | 95 (77.9%) | 522 | 324 (62.1%) | 98 | 52 (53.1%) |
| G3b | 2013 | 410 | 341 (83.2%) | 1377 | 969 (70.4%) | 226 | 123 (54.4%) |
| G4 | 1815 | 442 | 386 (87.3%) | 1224 | 973 (79.5%) | 149 | 105 (70.5%) |
| G5 | 333 | 88 | 74 (84.1%) | 217 | 189 (87.1%) | 28 | 23 (82.1%) |
| Not graded | 58 | 15 | 13 (86.7%) | 38 | 33 (86.8%) | 5 | 2 (40%) |

Treatment proportions calculated among patients with available hemoglobin measurements within each CKD stage and hemoglobin category. Percentages indicate proportion receiving ESA or HIF-PHI treatment within each subgroup.

ESA, erythropoiesis-stimulating agents; HIF-PHI, hypoxia-inducible factor prolyl hydroxylase inhibitors; Hb, hemoglobin; G1-G5, CKD stages based on eGFR categories according to KDIGO guidelines.
